# Quantum Hall states stabilized in semi-magnetic bilayers of topological insulators

**DOI:** 10.1038/ncomms9530

**Published:** 2015-10-26

**Authors:** R. Yoshimi, K. Yasuda, A. Tsukazaki, K. S. Takahashi, N. Nagaosa, M. Kawasaki, Y. Tokura

**Affiliations:** 1 Department of Applied Physics and Quantum-Phase Electronics Center (QPEC), University of Tokyo, 7-3-1Hongo, Bunkyo-ku, Tokyo 113-8656, Japan; 2 Institute for Materials Research, Tohoku University, Sendai 980-8577, Japan; 3 PRESTO, Japan Science and Technology Agency (JST), Chiyoda-ku, Tokyo 102-0075, Japan; 4 RIKEN Center for Emergent Matter Science (CEMS), Wako 351-0198, Japan

## Abstract

By breaking the time-reversal symmetry in three-dimensional topological insulators with the introduction of spontaneous magnetization or application of magnetic field, the surface states become gapped, leading to quantum anomalous Hall effect or quantum Hall effect, when the chemical potential locates inside the gap. Further breaking of inversion symmetry is possible by employing magnetic topological insulator heterostructures that host non-degenerate top and bottom surface states. Here we demonstrate the tailored-material approach for the realization of robust quantum Hall states in the bilayer system, in which the cooperative or cancelling combination of the anomalous and ordinary Hall responses from the respective magnetic and non-magnetic layers is exemplified. The appearance of quantum Hall states at filling factor 0 and +1 can be understood by the relationship of energy band diagrams for the two independent surface states. The designable heterostructures of magnetic topological insulator may explore a new arena for intriguing topological transport and functionality.

## Abstract (web summary)

The transport properties of materials with topologically non-trivial band structures may be manipulated by an applied magnetic field or by magnetic doping. Here, the authors demonstrate quantum Hall states for temperatures up to 2 K in thin film bilayers comprising pristine and Cr-doped topological insulators.

## Graphical abstract

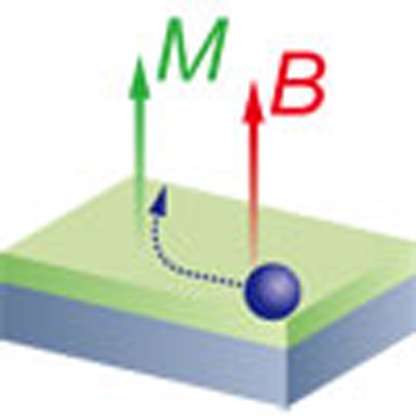

Three-dimensional (3D) topological insulator (TI) is a new class of material, which possesses an insulating bulk with a two-dimensional Dirac electron state on its surface^1^,^2^,^3^,^4^. When the time-reversal symmetry is broken with applying enough high magnetic fields or introducing spontaneous magnetization by doping magnetic impurities in 3D-TI, quantum Hall effect (QHE) or quantum anomalous Hall effect (QAHE) emerges as the hallmark of emergent states of two-dimensional electron system^5^. Recently the QHE and QAHE have been observed in 3D-TI thin films^6^,^7^ or cleaved bulk crystal^8^ and their magnetically doped compounds^9^,^10^,^11^. In spite of the large energy gaps due to the LL formation of the Dirac state (∼70 meV at *B*=14 T) (ref. 12) or the presence of the spontaneous magnetization (∼50 meV) (ref. 13), the observation of QHE or QAHE in the thin films has been limited so far at very low temperatures, typically below 100 mK, probably because the magnetic impurities and crystalline imperfections make the quantization difficult, owing to the level broadening.

In this study, we propose a magnetic TI system realizing the stable QH effect: the TI bilayer heterostructures composed of Cr-doped magnetic TI and pristine non-magnetic TI, hereafter referred to as semi-magnetic bilayers. From the theoretical^14^ and experimental^10^ points of view, the ground states of QHE and QAHE can be understood in the same context of topology. In the semi-magnetic TI bilayer, we can expect that the both magnetization *M* and magnetic field *B* identically drive the surface Dirac states in each TI layer to the QH states, which can be regarded as a hybrid phenomenon of QAHE in magnetic TI and QHE in non-magnetic TI layers with the common edge state. In other words, when the edge mode becomes stabilized, the system naturally converges to the QH state, yielding the quantized transverse conductivity *σ*_*xy*_∼*e*^2^*/h* (*e* and *h* are the elemental charge and Planck constant, respectively), irrespective of its origin. Moreover, as already suggested in the heterostructures with magnetic TI^15^ or non-TI magnetism^16^,^17^, the magnetic proximity effect on the non-magnetic TI surface from spatially separated magnetic layer may help the cyclotron gap or the exchange gap open much wider. Furthermore, the confinement of magnetic ions in a limited region of the heterostructure may suppress the disorder on the whole sample.

## Results

### Transport properties of semi-magnetic bilayers

**Figure 1:**
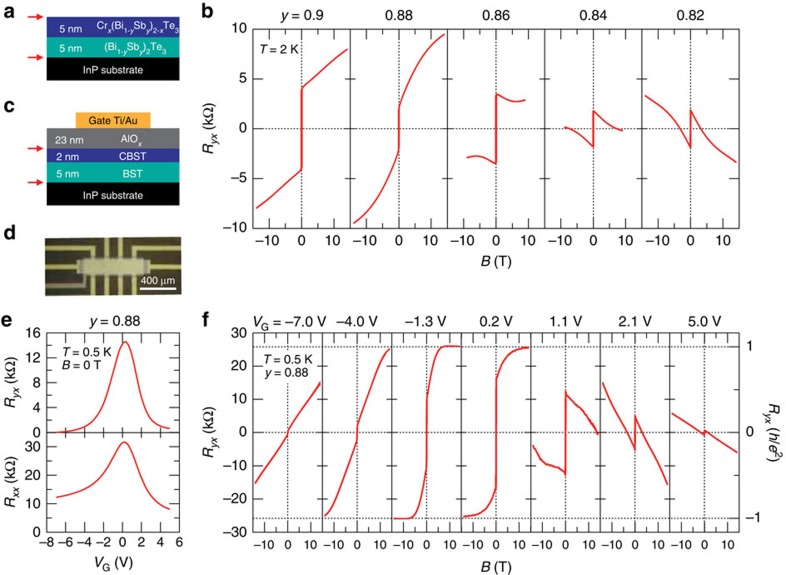
Hall responses *R*_*yx*_ in Cr_*x*_(Bi_1-*y*_Sb_*y*_)_2-*x*_Te_3_/(Bi_1-*y*_Sb_*y*_)_2_Te_3_ semi-magnetic TI bilayers. (**a**) A schematic of semi-magnetic TI bilayer composed of 5-nm CBST/5-nm BST. CBST and BST represent the Cr_*x*_(Bi_1−*y*_Sb_*y*_)_2−*x*_Te_3_ and (Bi_1−*y*_Sb_*y*_)_2_Te_3_, respectively. *x*-value is ∼0.2. Arrows indicate the interfaces where the Dirac state exists. (**b**) Transverse resistivity *R*_*yx*_ as a function of magnetic field *B* at *T*=2 K for several bare bilayers of 5-nm CBST/5-nm BST with different *y*. Cross-sectional schematic (**c**) and top-view photograph (**d**) of a FET with a Hall-bar channel of 2-nm CBST/5-nm BST (*x*∼0.2, *y*=0.88). Scale bar, 400 μm. (**e**) *V*_G_ dependence of *R*_*yx*_ and longitudinal resistivity (*R*_*xx*_) at *B*=0 T. (**f**) Magnetic field dependence of *R*_*yx*_ at *T*=0.5 K for several gate voltage *V*_G_ for FET device of 2-nm CBST/5-nm BST with *y*=0.88.

We fabricated TI bilayer heterostructures composed of Cr_*x*_(Bi_1−*y*_Sb_*y*_)_2−*x*_Te_3_ (CBST) and (Bi_1−*y*_Sb_*y*_)_2_Te_3_ (BST) on semi-insulating InP(111) substrates using molecular-beam epitaxy (MBE; see Supplementary Fig. 1 and Supplementary Note 1 for energy-dispersive X-ray spectroscopy mapping images of elements taken with a scanning transmission electron microscope), as schematically illustrated in Fig. 1a,c (see Supplementary Table 1 and Supplementary Note 2 for list of samples). Here we suppose the surface Dirac states should appear on the top surface of CBST as well as at the interface of BST and InP^12^, as indicated by red arrows, but not for the interface of TIs^18^,^19^. First, we investigate the Bi/Sb composition (*y*) dependence of *R*_*yx*_ in bare 5-nm CBST/5-nm BST heterostructures (Fig. 1a) with the same *y-*value to the both layers without AlO_*x*_ gate dielectric. Figure 1b shows the Hall resistance *R*_*yx*_ at *T*=2 K under magnetic field *B* for five samples with different *y*-values. We set the Cr concentration level (*x*) to 0.2 in this experiment. Although the anomalous Hall resistance at *B*=0 T is positive for all the samples, the slope of *R*_*yx*_ at high *B* region (ordinary Hall component) changes its sign depending on *y*, suggesting that charge-carrier type switches from hole to electron with decreasing *y*. These features resemble the case of the single layers of the BST and CBST, where it has been reported that Fermi level *E*_F_ can be tuned by the *y*-value in BST and CBST compounds^20^,^21^. As for the observed *R*_*yx*_ response, each BST and CBST layer appears to mainly contribute to the ordinary and anomalous Hall terms, respectively: a large value of 10 kΩ at *B*=14 T is observed for *y*=0.88, while a negative value for *y*=0.82. From these investigations on the 5-nm/5-nm heterostructures, we concluded that the *y*-values of 0.86 and of 0.88 represent the system with the *E*_F_ closest to the Dirac point and with the lowest hole density, respectively (see Supplementary Fig. 2 and Supplementary Note 3).

Next, to finely tune the *E*_F_ by electrical means, we defined a Hall-bar device for *y*=0.88 with AlO_*x*_ gate dielectric and Ti/Au gate electrode to fabricate field-effect transistor (FET) (see Methods). The schematic of vertical layered structure and top-view photographic image are shown in Fig. 1c,d, respectively. It is noteworthy that the thickness of CBST layer (*t*=2 nm) was optimized so as to exhibit the largest *R*_*yx*_ at high *B* corresponding to the lowest hole density (see Supplementary Fig. 2). *R*_*yx*_ and *R*_*xx*_ in Fig. 1e under the transistor operation at *T*=0.5 K with *B*=0 T show systematic changes as a function of *V*_G_; a single peak is observed at *V*_G_=0.2 V where both *R*_*yx*_ and *R*_*xx*_ reach the maximum, manifesting that the Fermi energy is close to the Dirac point. In Fig. 1f is shown the *B* dependence of *R*_*yx*_ measured with each *V*_G_ application. The *R*_*yx*_ slope at high *B* region varies systematically from positive to negative with the change of *V*_G_ from negative to positive, indicating the application of *V*_G_ effectively tunes *E*_F_ in the bilayer FET. As also shown in Fig. 1e,f, the anomalous Hall resistivity at *B*=0 T is conspicuously enhanced at around *V*_G_=−1.3 and 0.2 V; at these values of *V*_G_, the clear quantized Hall plateau of *R*_*yx*_ reaching *h*/*e*^2^=25.8 kΩ is observed on application of *B* with the large contribution of anomalous Hall term. In addition, the coercive field in *R*_*yx*_ hysteresis (see Supplementary Fig. 3 and Supplementary Note 4) is always observed in the present *V*_G_ range even in case of small *R*_*yx*_ response, indicating that the ferromagnetism survives when *E*_F_ is away from the Dirac point^9^,^10^,^21^.

### Quantum Hall states stabilized in semi-magnetic TI bilayers

**Figure 2:**
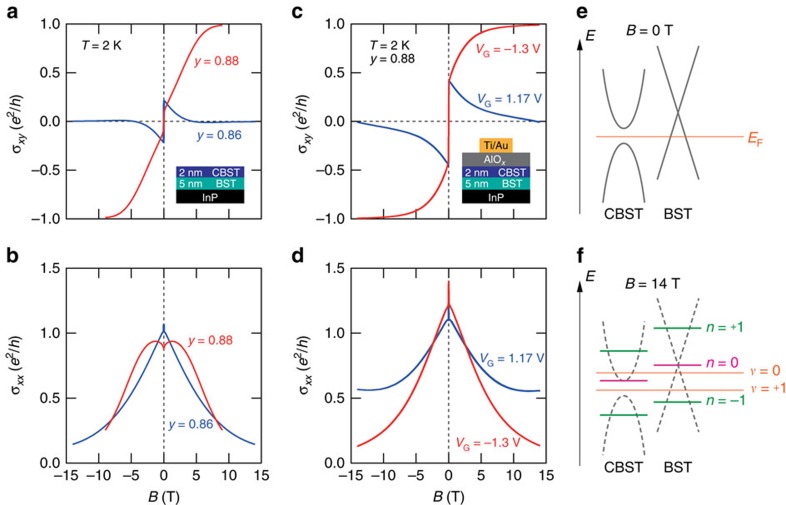
Conductivity responses in semi-magnetic TI bilayers observed at *T*=2 K. Magnetic field dependence of longitudinal and transverse conductivity *σ*_*xy*_ (**a**) and *σ*_*xx*_ (**b**) for *y*=0.88 and *y*=0.86 bare films of 2-nm CBST/5-nm BST at *T*=2 K. Magnetic field dependence of *σ*_*xy*_ (**c**) and *σ*_*xx*_ (**d**) for the FET device of *y*=0.88 at *V*_G_=−1.3 V (*ν*=+1) and *V*_G_=1.17 V (*ν*=0). (**e**,**f**) Schematic band diagram for the surface states of top CBST and bottom BST layers at magnetic field *B*=0 and 14 T, respectively. *E*_F_ represents the Fermi level at *V*_G_=0.2 V for the *y*=0.88 FET. In **f**, LLs *n*=+1, 0 and −1 are denoted by horizontal lines. Filling factor *ν* is indicated when *E*_F_ locates at the depicted energy position.

The feature of the QHE is also verified even at a higher temperature (for example, 2 K) for both bare films without gate structure and FET device. The *B* dependence of transverse and longitudinal conductivity *σ*_*xy*_ and *σ*_*xx*_ at *T*=2 K for the bare bilayers (2-nm CBST/5-nm BST) of *y*=0.88 (red) and 0.86 (blue) are displayed in Fig. 2a,b, respectively. For the *y*=0.88 bilayer film, *σ*_*xy*_ reaches the quantized value of *e*^2^/*h* accompanied by the decrease in *σ*_*xx*_ towards 0 with increasing *B*, which are a clear indication of the QH state at the filling factor of *ν*=+1. In the bilayer FET device of *y*=0.88, the similar behaviour of *ν*=+1 QH state is observed at *V*_G_=−1.3 V (red) as shown in Fig. 2c,d, with *σ*_*xy*_ of 0.988 *e*^2^/*h* and *σ*_*xx*_ of 0.134 *e*^2^/*h* at *B*=14 T in the FET device. In contrast, *σ*_*xy*_ for the bare bilayer film of *y*=0.86 and the *y*=0.88 bilayer FET at *V*_G_=1.17 V show the asymptotic behaviour towards 0 with increasing *B*, whereas *σ*_*xx*_ decreases similar to the case of the *ν*=1 QHE (blue) (down to *σ*_*xy*_=−0.002 *e*^2^/*h* and *σ*_*xx*_=0.144 *e*^2^/*h* at *B*=14 T in the bare bilayer film of *y*=0.86). We attributed this to the *ν*=0 QH state. In common for those two sample conditions, *E*_F_ is located above the Dirac point where electrons are dominant conduction carriers. The contribution of negative ordinary Hall term apparently cancels out the anomalous Hall term, resulting in the *ν*=0 state emerging at high *B*. A notable feature of these bilayer films is that the QH states, both *ν*=0 and *ν*=+1, are observable at 2 K, a much higher temperature than that of both QHE in BST^7^,^8^ and QAHE in CBST single-layer films^9^,^10^,^11^; this is true also in the case of bare films without *E*_F_ fine tuning.

## Discussion

The emergence of two QH states at *ν*=0 and +1 can be understood by the relationship of energy diagrams between the two independent surface bands of BST and CBST shown in Fig. 2e,f. First of all, we confirm that neither QHE nor QAHE is observed in single layer films of 5-nm BST and 2-nm CBST (see Supplementary Fig. 4 and Supplementary Note 5), implying no surface Dirac states formed due to hybridization between two surfaces in thin single layers. We focus on the energy relationship of the two surface states in bilayer between the magnetization-induced gap on the top surface of CBST layer and the Dirac point on the bottom surface of BST layer. When *V*_G_ of 0.2 V is applied, *E*_F_ locates around the centre of the gap of CBST surface state, as anomalous Hall term in *R*_*yx*_ at *B*=0 T reaches maximum, as shown in Fig. 1e. With applying magnetic field, the ordinary Hall term is added to the anomalous Hall term. The observed positive ordinary Hall term at *V*_G_=0.2 V shown in the centre panel of Fig. 1f indicates that *p*-type carrier dominantly comes from the bottom BST surface state, because the top CBST surface state with *E*_F_ within the gap should minimally contribute to the ordinary Hall term. Therefore, the relative energy position around the Dirac point between CBST and BST surfaces at *V*_G_=0.2 V under *B*=0 T is as depicted in Fig. 2e. Under high magnetic field, the LLs are formed from the Dirac band dispersion as schematically illustrated in Fig. 2f. Here, *n*=0 LL forms on one side of the massive Dirac cone depending on a sign of the mass term of TI. In case of CBST, it is known to form at the bottom of higher energy one^21^. When *E*_F_ is below the *n*=0 LLs of both top and bottom surfaces, the *v*=+1 QH state emerges. Following the band relationship shown in Fig. 2f, fine tuning of *E*_F_ between the two *n*=0 LLs enables us to achieve the *ν*=0 state.

**Figure 3:**
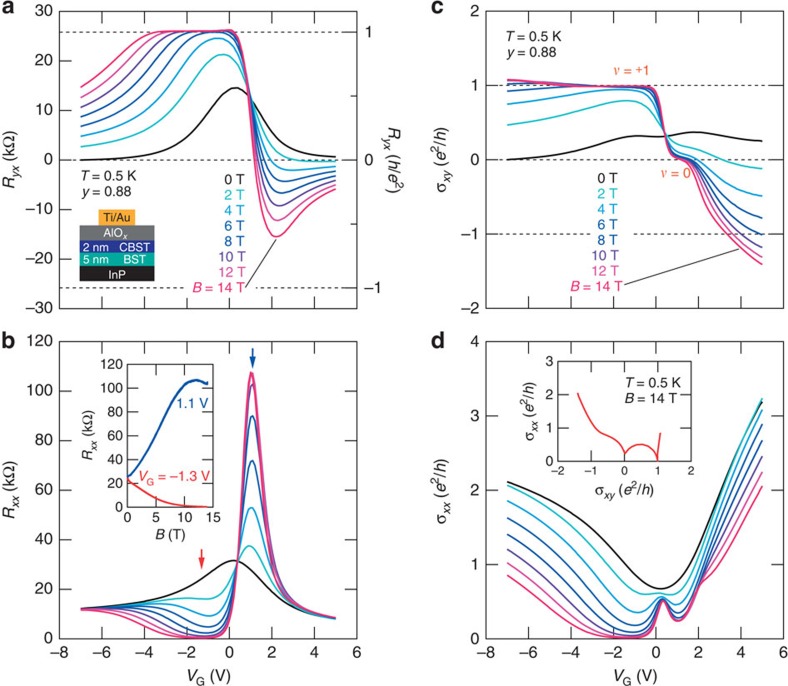
Magnetic field dependence of QH states in gate-tuned semi-magnetic TI bilayers. *V*_G_ dependence of *R*_*yx*_ (**a**) and *R*_*xx*_ (**b**) for *y*=0.88 FET at *T*=0.5 K under various magnetic fields. The inset in **b** shows the magnetic field dependence of *R*_*xx*_ at *V*_G_=1.1 V (blue) and *V*_G_=−1.3 V (red). Blue and red arrows in the main panel of **b** represent gate voltages of *V*_G_=1.1 and −1.3 V, respectively. *V*_G_ dependence of *σ*_*xy*_ (**c**) and *σ*_*xx*_ (**d**) at *T*=0.5 K under various magnetic fields. The inset in **d** plots the (*σ*_*xy*_(*V*_G_), *σ*_*xx*_(*V*_G_)) at various *V*_G_ under magnetic field *B*=14 T.

To identify *E*_F_ location in the two surface bands drawn in Fig. 2e,f more precisely, *V*_G_ control measurements for *R*_*xx*_ and *R*_*yx*_ are performed. The QHE in FET is clearly demonstrated at various magnetic fields in Fig. 3a–d. Hallmarks of *ν*=+1 are observed at around *V*_G_=−1.3 V (red arrow in Fig. 3b) with increasing *B*; *R*_*yx*_∼25.8 kΩ (Fig. 3a), *σ*_*xy*_=+*e*^2^/*h* (Fig. 3c) and *R*_*xx*_ and *σ*_*xx*_∼0 (Fig.3b,d). The other QH plateau at *v*=0 in *σ*_*xy*_ (Fig. 3c) is realized around *V*_G_=1.1 V, *R*_*yx*_∼0 (Fig. 3a) and high *R*_*xx*_ (blue arrow in Fig. 3b). Correspondingly, *R*_*xx*_ in the inset of Fig. 3b increases up to about 100 kΩ with increasing *B* to 14 T. In this case, positive anomalous and negative ordinary Hall resistivity almost cancels out, resulting in a small value of total *R*_*yx*_. In addition, a dip in *σ*_*xx*_ versus *V*_G_ curve is simultaneously obtained at *V*_G_=1.1 V in Fig. 3d. This exemplifies the *ν*=0 pseudo-spin Hall insulator state^7^, in which we view the top and bottom degrees of freedom in the surface states as the pseudo spins, as expected from band diagram shown in Fig. 2f. The appearance of *ν*=0 in *σ*_*xy*_ at high *B* between two peaks of *σ*_*xy*_ at *B*=0 T (black curve in Fig. 3c) is a compelling evidence for the *E*_F_ location as discussed above. Indeed, the QH states *ν*=0 and +1 can be clearly resolved in the conductivity mapping (*σ*_*xy*_ (*V*_G_), *σ*_*xx*_ (*V*_G_)) at various *V*_G_, as shown in the inset of the Fig. 3d.

Finally, we discuss why the QH state in the bilayers of BST and CBST are observable up to higher temperatures or much more stable than those of single-layer films of BST and CBST. We raise two possible reasons: the one is a magnetic proximity effect on the BST surface from the adjacent ferromagnetic CBST layer and the other is less disorder at the surface conduction channels in the present bilayer setup. Regarding the proximity effect, the increase in Hall response with application of magnetic field at negative *V*_G_ (hole accumulation) is approximately three times as large as that at positive *V*_G_ (electron side) (see Supplementary Fig. 5). This enhancement of Hall response at negative *V*_G_ coincides with the peak of anomalous *σ*_*xy*_ response (*B*=0 T, black curve in Fig. 3c) at *V*_G_=−1.3 V, pointing to the proximity effect from the magnetic CBST layer to the BST surface state (see also Supplementary Note 6), which leads to the observation of QHE at high temperature compared with the single-layer FETs of BST (normal QHE)^7^ and CBST (QAHE)^9^,^10^,^11^. It is worth noting here that we do not explicitly consider the magnetizatioin-induced gap opening at *B*=0 T in BST layer by the proximity effect, while the anomalous Hall term is enhanced at finite *B*. Second, as for the possible effect of disorder, the interface combinations of AlO_*x*_/CBST and BST/InP may have less disorder than those of AlO_*x*_/BST and CBST/InP; this is speculated from the small Hall response of the semi-magnetic heterostructure with the inverted structure of BST/CBST/InP (see Supplementary Figs 6–8 and Supplementary Note 7). These two effects may work cooperatively to stabilize the QHE states in the semi-magnetic bilayers.

In conclusion, we have successfully resolved the QH states in semi-magnetic bilayers of TI. The surface state readily exhibits the *ν*=0 and +1 QH states under a relatively small magnetic field with a large contribution from the anomalous Hall term in magnetic TI layer. These QH states are accounted for in terms of the magnetization-induced gap and/or the formation of LLs at each component-layer surface state. Furthermore, the observation at a relatively high temperature (*T*=2 K) suggests the semi-magnetic structure may have the proximity effect, while suppressing the disorder effect for surface transport. TI-based semi-magnetic heterostructures and superlattices may provide a new platform in exploring new functionality and exotic phases of TIs^22^,^23^.

## Methods

### MBE thin film growth

Bilayer thin films of BST and CBST were fabricated by MBE on semi-insulating InP (111) substrates. The Bi/Sb composition ratio *y* for bare films was calibrated by the beam equivalent pressure of Bi and Sb, for example, 6 × 10^−7^ and 4.4 × 10^−6^ Pa for *y*=0.88. As the formation of AlO_*x*_/Ti/Au gate structure changes the *E*_F_ position in the channel, we fabricated FETs with various *y*-values close to 0.88 for maximizing *R*_*yx*_ at *V*_G_=0 V under large *B*. The designed *y*-value for the FET channel was 0.84, but from the comparison with the transport properties of the bare films the *y*-value is indicated as 0.88 in this study for the sake of consistency and readability. The Cr concentration *x* was determined by the flux ratio of Cr/(Bi+Sb). The Te flux was overssupplied with keeping the Te/(Bi+Sb) ratio at about 20. Before the growth of the first layer of BST, we started with supplying Te and Sb for a growth of monolayer Sb_2_Te_3_ buffer layer to construct a smooth interface with InP substrate.

### FET device fabrication

After the epitaxial growth of BST layer, films were annealed *in situ* at 380 °C, to make the surface smoother under the exposure of Te flux. The same procedure was employed for the following CBST layer. For the preparation of FET devices, AlO_*x*_ capping layer was deposited at room temperature with an atomic layer deposition system immediately after the discharge of the samples from MBE. The device pattern was defined by photolithography and Ar ion-milling processes. Here, ion-milling was performed under 45° tilt condition on a rotating stage, resulting in the ramped side edge. This ensured electrical contact to both the top and bottom of the film.

### Transport measurements

Ohmic-contact electrodes and top gate electrode were Ti/Au deposited with an electron-beam evaporator. Transport measurements for bare films were conducted using the d.c. transport option of physical property measurement system (PPMS) by Quantum Design. FET devices were measured in PPMS with employing a lock-in technique at a frequency (∼3 Hz) and with a low excitation current (∼ 10 nA) to suppress heating effect. A series resistance of 100 MΩ was introduced to maintain a constant current condition. Low temperature (<2 K) measurements were performed using the ^3^He option of PPMS.

## Additional information

**How to cite this article:** Yoshimi, R. *et al.* Quantum hall states stabilized in semi-magnetic bilayers of topological insulators. *Nat. Commun.* 6:8530 doi: 10.1038/ncomms9530 (2015).

## Supplementary Material

Supplementary Information Supplementary Figures 1-8, Supplementary Table 1, Supplementary Notes 1-7 and Supplementary References
