# Supplementary material for: Quantum Hall states stabilized in semi-magnetic bilayers of topological insulators
Source: Nat Commun. 2015 Oct 26;6:8530. doi: 10.1038/ncomms9530 (PMC4639800; doi:10.1038/ncomms9530)
Supplement: Supplementary Information — Supplementary Figures 1-8, Supplementary Table 1, Supplementary Notes 1-7 and Supplementary References [file ncomms9530-s1.pdf]

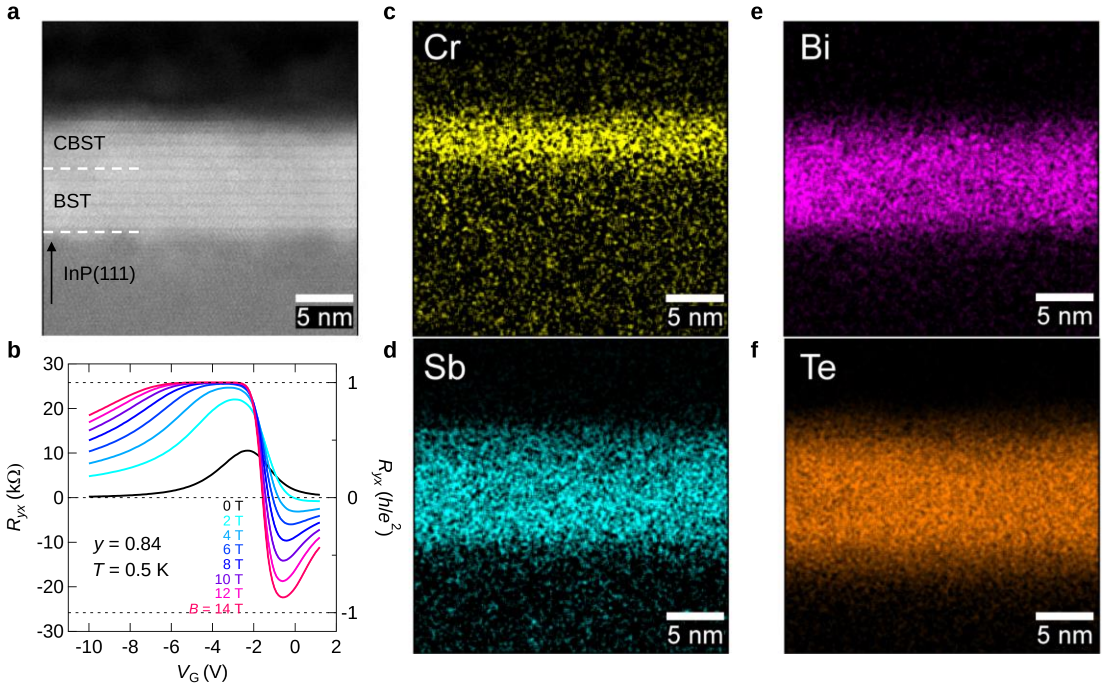

Supplementary Figure 1 (a) Scanning transmission electron microscope image of bilayer sample (b) Hall resistance of the sample shown in (a) as a function of V_G under magnetic field. (c-f) Distribution maps of (c) Cr, (d) Sb, (e)Bi, (f) Te taken by energy-dispersive x-ray spectroscopy.

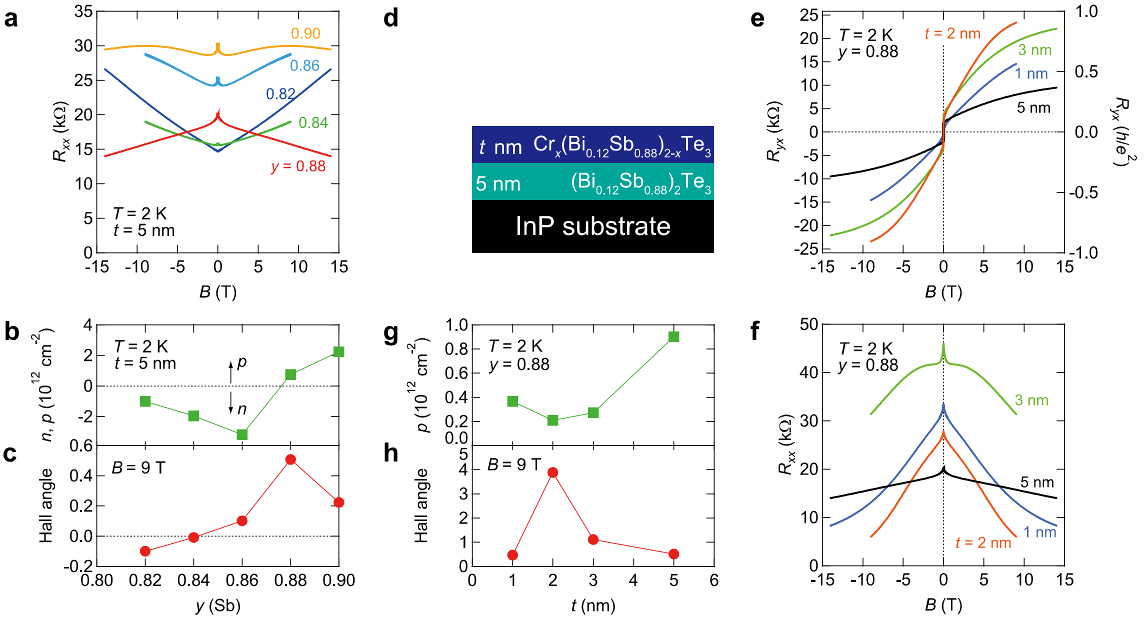

Supplementary Figure 2 (a) Longitudinal resistivity R_{xx} as a function of magnetic field B in bilayers with the 5-nm-thick CBST/5-nm BST structure for five y values, simultaneously measured with R_{yx} shown in Fig. 1b. **(b, c)** Bi/Sb composition ratio y dependence of carrier density (b) and Hall angle at B = 9 T (c). **(d)** A schematic view of semi-magnetic bilayer with t-nm-thick CBST/5-nm BST. **(e, f)** B dependence of R_{yx} (e) and R_{xx} (f) in bilayers with y = 0.88 for different CBST layer thicknesses. **(g, h)** CBST layer thickness t dependence of the hole density p (g) and Hall angle at B = 9 T (h).

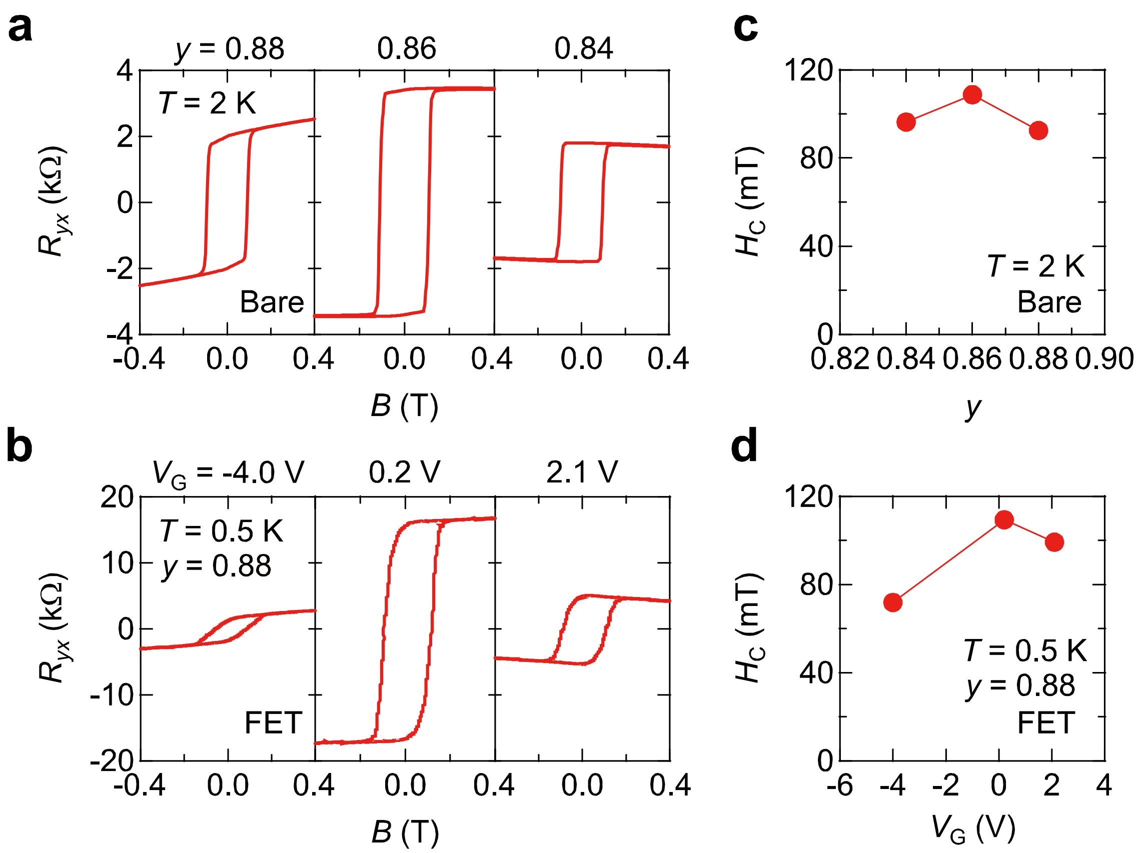

Supplementary Figure 3 (a) Anomalous Hall effect at low magnetic field for bare films with y = 0.88, 0.86 and 0.84 (No. 2, 3 and 4 in Supplementary Table 1). **(b)** Anomalous Hall effect for a FET device (No. 8 in Supplementary Table 1) with gate voltages V_G = −4.0, 0.2, and 2.1 V. **(c)** y dependence of coercive field H_C in 5-nm CBST/5-nm BST/InP. **(d)** V_G dependence of H_C in y = 0.88 FET device.

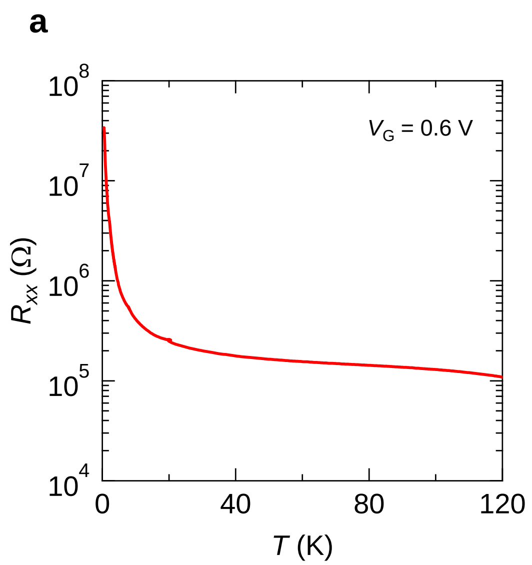

Supplementary Figure 4 (a) Temperature dependence of resistance in 5 nm BST thin film. **(b)** Gate voltage dependence of longitudinal resistance R_{xx} at T = 0.5 K under B = 0 and 14 T.

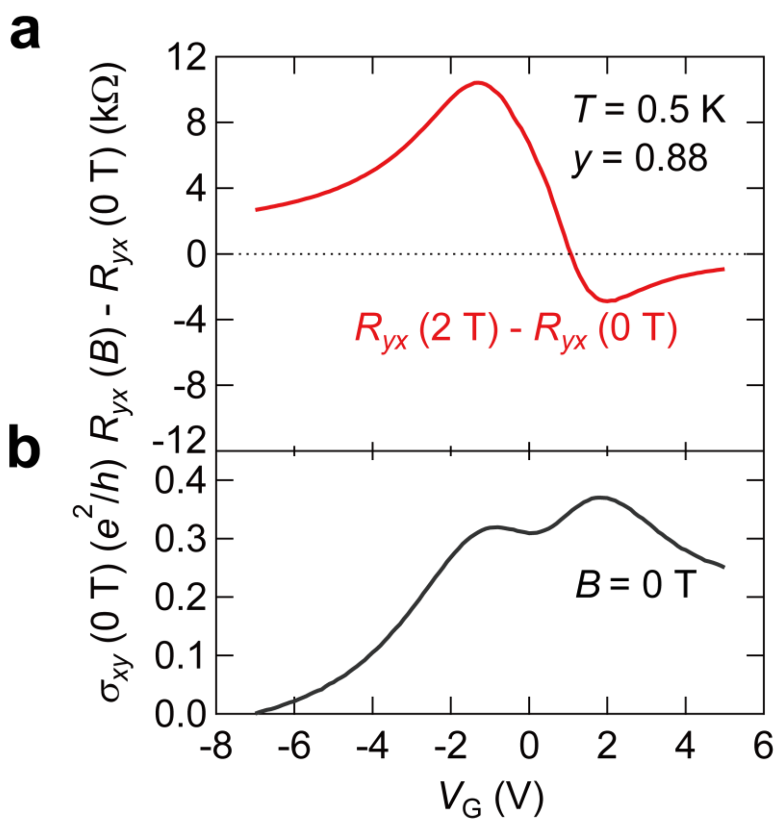

Supplementary Figure 5 (a) The magnetic field induced Hall response in y = 0.88 FET device under B = 2 T, calculated from the subtraction of R_{yx} at 0 T from that at 2 T. **(b)** Anomalous Hall conductivity σ_{xy} at B = 0 T as a function of V_G.

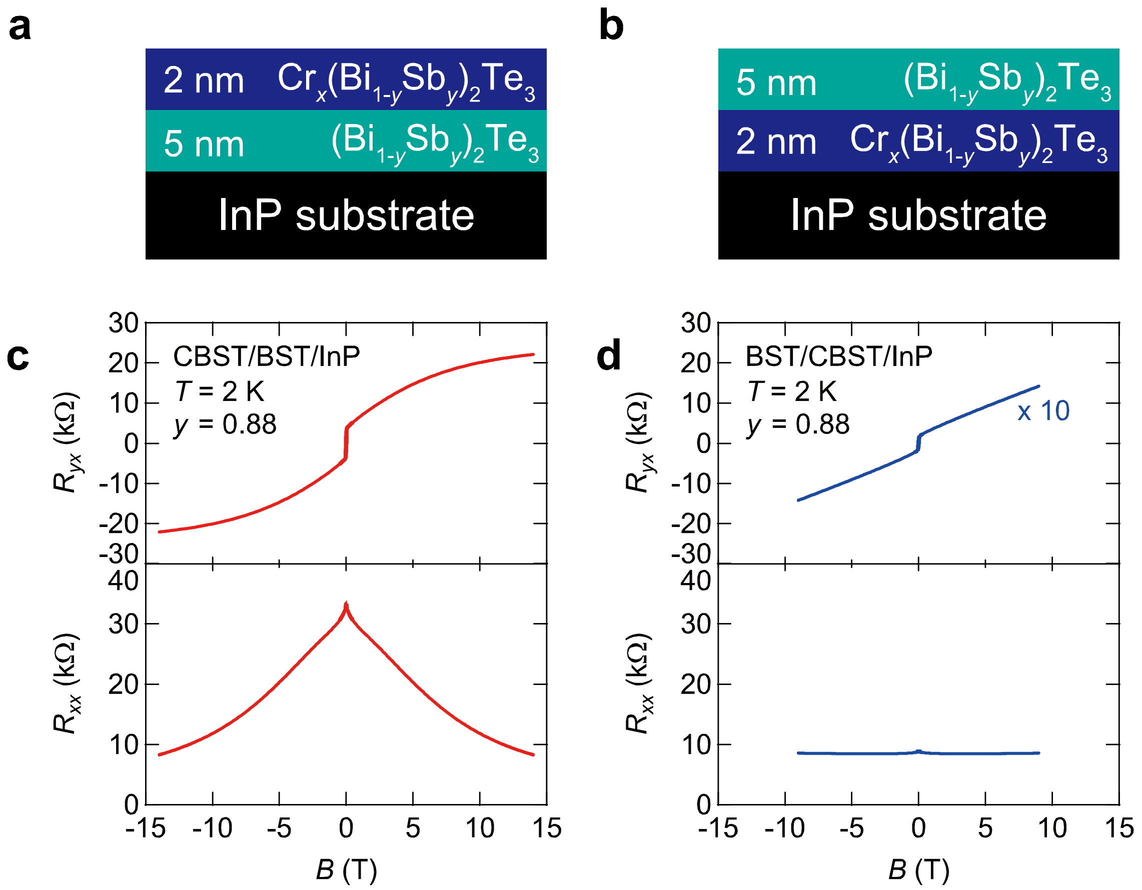

Supplementary Figure 6 (a, b) Cross-sectional schematics of bilayers, the normal CBST/BST/InP structure discussed in the main text (a), and the inverted BST/CBST/InP structure (b). **(c, d)** The B dependence of R_{yx} and R_{xx} for each structure. (c) is for CBST/BST/InP and (d) is for BST/CBST/InP.

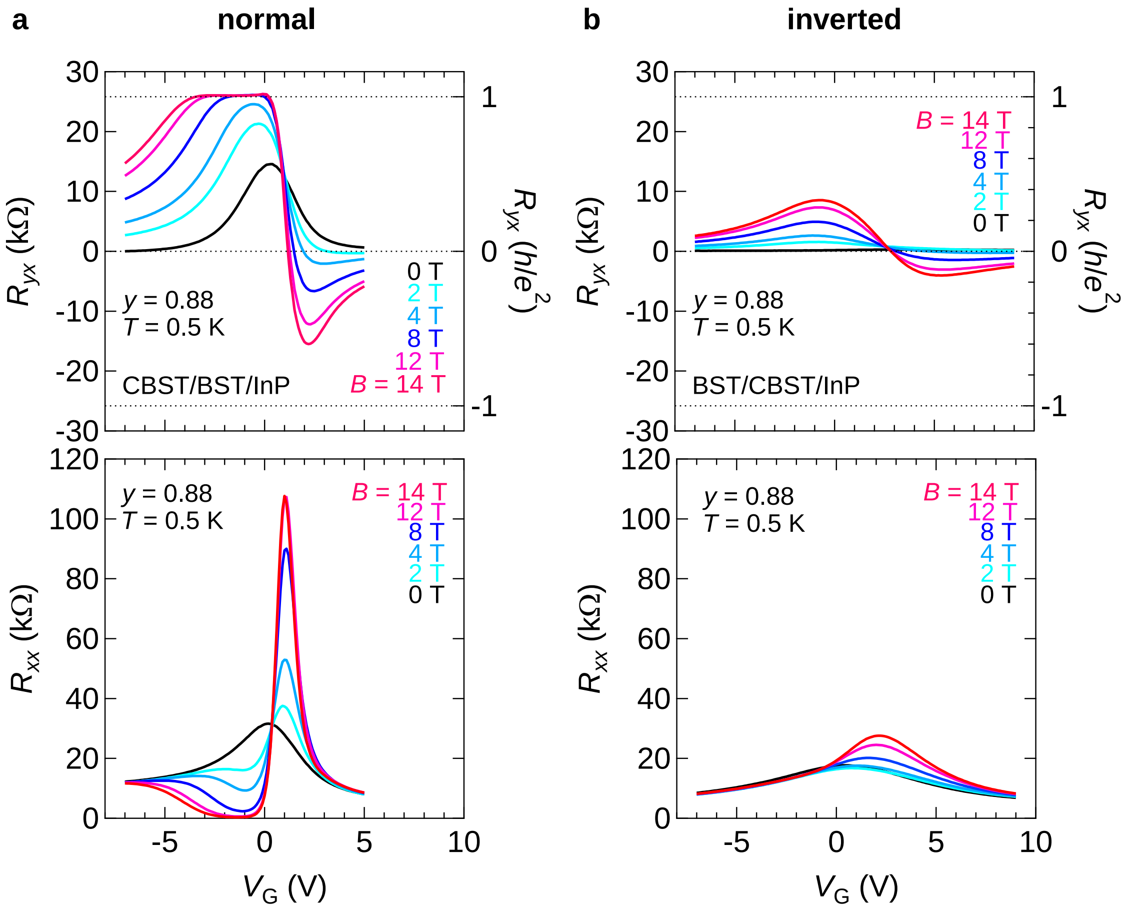

Supplementary Figure 7 Hall resistance R_{yx} and longitudinal resistance R_{xx} as a function of gate voltage at T = 0.5 K in CBST/BST/InP normal structure (a) and BST/CBST/InP inverted structure (b) for several magnetic fields.

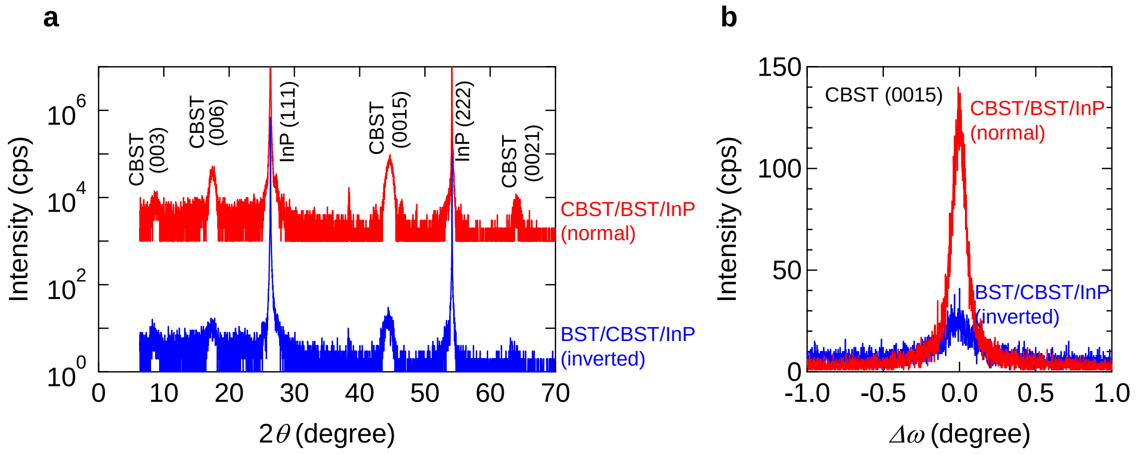

Supplementary Figure 8 **(a)** X-ray diffraction patterns for CBST/BST/InP normal structure (red) and BST/CBST/InP inverted structure (blue). Data for normal structure is shifted for clarity. **(b)** Rocking scan around CBST (0015) peak.

Supplementary Table 1 | Parameters of bilayer heterostructures discussed in main text.

No.	Figure	*y*	Bare or FET	Thickness of CBST layer (nm)	Thickness of BST layer (nm)
1	Fig. 1b	0.90	Bare	5	5
2	Fig. 1b	0.88	Bare	5	5
3	Fig. 1b	0.86	Bare	5	5
4	Fig. 1b	0.84	Bare	5	5
5	Fig. 1b	0.82	Bare	5	5
6	Fig. 2a, 2b	0.88	Bare	2	5
7	Fig. 2a, 2b	0.86	Bare	2	5
8	Fig. 1e, 1f, 2c, 2d, 3a-d	0.88	FET	2	5

Supplementary Note 1 | STEM-EDX measurement on a CBST/BST/InP semi-magnetic bilayer

Supplementary Fig. 1a shows the cross sectional TEM image of bilayer sample CBST/BST/InP whose x(Cr) and y(Sb) are set to 0.2 and 0.84, respectively. The ordered epitaxial layers of BST and CBST on InP(111) are well resolved. Here, the quantum transport is reproduced in this sample as shown in Supplementary Fig. 1b. This sample basically shows similar transport properties to those shown in Fig. 3a in the main text with $\nu = +1$ quantum Hall plateau, ensuring the validity of our TEM measurements for the samples in the main text. The distributions of respective elements, Cr, Sb, Bi and Te, in the bilayer shown in Supplementary Fig. 1a are illustrated in Supplementary Fig. 1c-1f. Although those for Bi, Sb and Te look homogeneously distributed over the whole layers, Cr is concentrated only on the designed top CBST layers, which evidences that we could fabricate bilayers of CBST/BST without interdiffusion of Cr.

Supplementary Note 2 | The list of samples and their correspondence to the Figures in the main text

Supplementary Table 1 summarizes the samples used in this study and indicates the Figures involving the corresponding data. The transport measurements were conducted for bare films and a FET device. In Fig. 1b, R_{yx} for 5 bare bilayers with different y values are displayed (No. 1-5 in the Supplementary Table 1), whose structure is 5-nm CBST/5-nm BST/InP. Bare bilayer films of 2-nm CBST/5-nm BST/InP structure with y = 0.88 (No. 6) and 0.86 (No. 7) are shown in Fig. 2a and 2b. All the FET data are taken for a FET device with y = 0.88 and 2-nm CBST/5-nm BST/InP structure (No. 8).

Supplementary Note 3 | Optimization of Bi/Sb composition ratio and CBST layer thickness

In order to maximize R_{yx} in bilayer system, we first optimized the Bi/Sb composition y in 5-nm CBST/5-nm BST structure (Fig. 1a and 1b in the main text) and then tuned CBST layer thickness t as schematically illustrated in Supplementary Fig. 2d. B dependence of R_{xx} is shown in Supplementary Fig. 2a with different y values (sample No. 1-5 in Supplementary Table 1). Only y = 0.88 sample shows R_{xx} monotonically decreasing with increasing B, while other samples show positive magnetoresistance or weak B dependence (y = 0.90). Accompanied with negative magnetoresistance, magnetic field induced R_{yx} defined as R_{yx} (B) − R_{yx} (0 T) in y = 0.88 is the largest as shown in Fig. 1b in the main text, which results in the lowest hole density as shown in Supplementary Fig. 2b. Note that the charge carrier density is evaluated by the slope of R_{yx} at low B region. Owing to the small R_{xx} and large R_{yx}, the largest Hall angle at B = 9 T defined as R_{yx}/R_{xx} is attained at y = 0.88 as shown in Supplementary Fig. 2c. From these results, we fix the y value at 0.88 for the examination of thickness t dependence for the samples indicated in Supplementary Fig. 2d. Supplementary Figs. 2e and 2f show the R_{yx} and R_{xx}, respectively, for y = 0.88 bilayers with different CBST layer thickness t. The behavior of increasing R_{yx} and decreasing R_{xx} with increasing B is displayed for all the samples. Hole density and Hall angle are calculated in the same way as y dependence discussed in Supplementary Figs. 2b and 2c and are displayed in Supplementary Figs. 2g and 2h, respectively. From the smallest hole density and largest Hall angle, the optimum thickness is found out to be 2 nm.

Supplementary Note 4 | R_{yx} at low magnetic field

Supplementary Figures 3a and 3b show the B dependence of R_{yx} in the bare 5-nm CBST/5-nm BST bilayer films of y = 0.88, 0.86 and 0.84 and a FET device of y = 0.88. R_{yx} data up to 14 T are shown in Figs. 1b and 1e in the main text, respectively. The clear hysteresis loops indicate spontaneous ferromagnetism. The coercive field H_C of bare films and a FET device are displayed in Supplementary Figs. 3c (bare) and 3d (FET). While y dependence or V_G dependence of H_C is not clear in this experiment, finite H_C exemplifies the ferromagnetism in the bilayer.

Supplementary Note 5 | Transport properties of single layer films of 5-nm BST and 2-nm CBST

No QH states are observed in sole film of 5 nm BST or 2 nm CBST. Supplementary Figs. 4a and 4b show the transport property in 5 nm BST thin film. The resistivity increases with decreasing temperature as shown in Supplementary Fig. 4a, exemplifying the insulating behavior of the thin film. At lowest temperature of T = 0.5 K, the gate voltage dependence under the magnetic fields of 0 T and 14 T in Supplementary Fig. 4b shows that R_{xx} reaches above 100 kΩ in wide range of the gate voltage, indicating that neither QAHE nor QHE is observed. Here, we cannot measure the Hall resistance precisely, because R_{xx} is so large that we cannot neglect its contribution to Hall resistance coming from the small displacement of Hall voltage probe even in the form of well-defined Hall bar. Furthermore, the peak of R_{xx} with significant high resistance over 1 MΩ between 0 and 1.5 V suggests that in-gap Dirac states is no more gapless. 5 nm is too thin to form the gapless surface states due to hybridization between two surface states. As for the 2 nm CBST film, the situation is similar. In that case, the resistance is over 10 MΩ even at room temperature, showing the film is completely insulating. From these results, in the sole films of 2 nm CBST and 5 nm BST, we could confirm that neither the QHE nor the QAHE is observed, possibly owing to the interplay of top and bottom Dirac states, which opens a gap in Dirac state.

In the bilayer system, however, the situation is different. Because this heterostructure is a junction of two TIs, there is no Dirac state at the interface of two TIs [1]. Therefore, what should be considered is the interplay between the top of CBST and bottom of BST layer. In the present case, whole thickness is about 7 nm, which is thick enough to neglect the hybridization between two surfaces. If the total thickness is less than critical value of approximately 5 nm [2], QHE cannot be observed due to the opening of the hybridization gap. Furthermore, the quantized Hall conductance of $\sigma_{xy} = e^2/h$ observed in our experiment is the evidence of the existence of two Dirac states, because each Dirac state has the contribution of $e^2/2h$.

Supplementary Note 6 | Enhancement of Hall response under magnetic field

The increase in Hall response with applying B is examined by subtracting the anomalous Hall term R_{yx} at B = 0 T, namely $R_{yx}(B) - R_{yx}(0\ \mathrm{T})$. Supplementary Figure 5a displays the magnetic field induced R_{yx} under a low B = 2 T, where no quantization feature appears due to the low mobility. The positive and negative peaks appear at around V_{G} = −1.3 V and +2.0 V, corresponding to hole and electron accumulation, respectively. The peak at hole accumulation side is three times larger than that at electron side, indicating that Hall response is more pronounced in the hole accumulation side. It may originate from the energy band features such as difference in Fermi velocity between electron and hole side, and proximity effect of ferromagnetic CBST to BST as speculated in the main text.

Supplementary Figure 5b shows the anomalous Hall conductivity σ_{xy} at B = 0 T, calculated by R_{yx} and R_{xx} shown in Fig. 1e in the main text. Although R_{yx} and R_{xx} show almost symmetric behavior against V_{G} variation (Fig. 1e), two peaks appear in σ_{xy} at V_{G} = −1.0 V and +1.8 V as shown in Supplementary Fig. 5b, which coincide with the positive and negative peaks in R_{yx} displayed in Supplementary Fig. 5a. This indicates that anomalous Hall conductivity gives maximum at two different positions of Fermi energy in the bilayer system. In single layer FET based on BST [3] and CBST [4, 5], σ_{xy} shows single peak at QHE under high B and QAHE condition at zero magnetic field. Therefore, among three possible origins raised in the previous paragraph, proximity effect of magnetic feature in CBST interacting with surface charge carrier at BST is the most plausible explanation. These results could be a compelling signature for the cooperative interaction of two surfaces, clarifying potential advantage in asymmetric TI bilayers.

Supplementary Note 7 | Transport properties of bilayers with inverted structure

The disorder at the surface or interface is examined with inverted structure. Supplementary Figures 6a and 6b illustrate two types of bare bilayer structures, normal CBST/BST/InP and inverted BST/CBST/InP, respectively. Here, thickness of each layer and Bi/Sb composition ratio y are same. The transport properties of these structures are displayed in Supplementary Figs. 6c and 6d. The normal CBST/BST/InP structure shows a large enhancement in R_{yx} and negative magnetroresistance, resulting in large Hall angle and QHE. However, R_{yx} in inverted BST/CBST/InP structure gives an order smaller R_{yx} than normal CBST/BST/InP structure. R_{xx} in the inverted structure has almost no filed dependence. Furthermore, we investigated the gate voltage dependence of R_{yx} and R_{xx} in field-effect transistor (FET) on the inverted bilayer as shown in Supplementary Figure 7b, in order to compare with Supplementary Figure 7a which is the same data set shown in Fig. 3a and 3b in the main text. Although Fermi level (E_F) in the inverted bilayer FET is tuned across Dirac point (DP) via gating, R_{yx} maxima did not reach the quantum value of 1 h/e^2 either at both *p*- or *n*-type regions, implying no stabilization effect took place. In addition, the maximum of anomalous Hall response at 0 T is tiny in the inverted structure probably due to weak ferromagnetic order and/or large disorder in potential around E_F in 2-nm CBST. Therefore, we conclude that the stacking sequence plays a key role to induce stabilization of quantum states in bilayer systems. The much smaller Hall response in the inverted BST/CBST/InP structure indicates that interface state of CBST/InP is more disordered than that of BST/InP as discussed below.

Supplementary Figure 8 shows the X-ray diffraction patterns for CBST/BST/InP (normal) and BST/CBST/InP (inverted) structures. All peaks in Supplementary Fig. 8a can be assigned as (0 0 0 *n*) diffractions of BST (CBST) or (*n n n*) ones of InP for both normal and inverted cases. In the rocking scan around the (0 0 0 15) peaks shown in Supplementary Fig. 8b, however, the intensity and sharpness of the peaks are different in two structures. The CBST/BST/InP normal structure exhibits sharper rocking curve than BST/CBST/InP inverted structure, indicating better coherence between layers. Therefore, we conclude that the poor transport property originates from the worse crystal quality of the inverted structure.

Supplementary References

1. Zhao, Y., et al., Demonstration of surface transport in a hybrid Bi2Se3/Bi2Te3 heterostructure. *Sci. Rep.* **3** 3060 (2013).
2. Zhang, Y. *et al.* Crossover of the three-dimensional topological insulator Bi_2Se_3 to the two-dimensional limit. *Nature Phys.* **6**, 584-588 (2010).
3. Yoshimi, R., *et al.*, Quantum Hall Effect on Top and Bottom Surface States of Topological Insulator $(Bi_{1-x}Sb_x)_2Te_3$ Films. *Nature Commun.* **6** 6627 (2015).
4. Chang, C. –Z., *et al*., Experimental Observation of the Quantum Anomalous Hall Effect in a Magnetic Topological Insulator. *Science* **340**, 167-170 (2013).
5. Checkelsky, J. G. *et al.*, Trajectory of the anomalous Hall effect towards the quantized state in a ferromagnetic topological insulator. *Nature Phys.* **10**, 731-736 (2014).
